# Learning directed acyclic graphs from large-scale genomics data

**DOI:** 10.1186/s13637-017-0063-3

**Published:** 2017-09-20

**Authors:** Fabio Nikolay, Marius Pesavento, George Kritikos, Nassos Typas

**Affiliations:** 1 Communication Systems Group, TU Darmstadt, Merckstr. 25, Darmstadt, Germany; 2 European Molecular Biology Laboratory, Heidelberg, Meyerhofstraße 1, Heidelberg, 69117 Germany

**Keywords:** Genetic interaction analysis, Large-scale gene networks, Discrete optimization, Graph learning, Big data, Multiple hypothesis test

## Abstract

In this paper, we consider the problem of learning the genetic interaction map, i.e., the topology of a directed acyclic graph (DAG) of genetic interactions from noisy double-knockout (DK) data. Based on a set of well-established biological interaction models, we detect and classify the interactions between genes. We propose a novel linear integer optimization program called the Genetic-Interactions-Detector (GENIE) to identify the complex biological dependencies among genes and to compute the DAG topology that matches the DK measurements best. Furthermore, we extend the GENIE program by incorporating genetic interaction profile (GI-profile) data to further enhance the detection performance. In addition, we propose a sequential scalability technique for large sets of genes under study, in order to provide statistically significant results for real measurement data. Finally, we show via numeric simulations that the GENIE program and the GI-profile data extended GENIE (GI-GENIE) program clearly outperform the conventional techniques and present real data results for our proposed sequential scalability technique.

## Introduction

Genetic interaction analysis aims at uncovering the interactions among a set of genes with respect to a specified cell function of a biological system, e.g., the fitness of a specific bacteria colony. The interactions among the genes under study can be characterized by a directed acyclic graph (DAG) [1] where the hierarchical relationship among two genes of a DAG describes their hierarchical interaction type [2]. However, DAGs cannot be observed directly but only the specified cell function under study which yields observable phenotypes. The term phenotype generally describes the specific manifestation of a biological attribute of an organism which can be observed, e.g., for bacteria, a common biological attribute is the growth measured in colony size, where a specific size of the bacteria colony is a phenotype of this biological attribute.

The role of the studied genes in the cell machinery and the hierarchical interaction types of the genes, as well as the DAG, which describes the latter ones, can only be learned by means of knockout experiments where a gene or a set of genes is functionally switched off and the phenotype is observed. Traditionally, only single-knockout (SK) experiments have been conducted but those mainly provide evidence on the importance of a single gene for the investigated cell process and do not convey much information about the interaction among the genes under study.

Recently, with the technological advances in microarrays and the development of the synthetic genetic array technologies [3], new approaches have been taken that are based on large-scale knockout experiments of pairs of genes. Such double-knockout (DK) experiments are much more powerful for exploring genetic interactions since a DK phenotype of an arbitrary pair of genes generally differs considerably from the superposition of the corresponding SK phenotypes of this pair of genes. According to [2], the gene pairs can be classified into one out of five hierarchical relationship classes based on their SK and DK phenotypes. Further, based on the hierarchical relationship classes, the DAG underlying the observed SK and DK phenotypes can be inferred which directly reflects the genetic interactions among the genes.

In order to detect the DAG underlying the SK and DK phenotypes, a variety of statistical methods based on scoring the measurements or on thresholding the genetic interaction (GI)-profile data, which is commonly based on Pearson correlation of the SK and DK phenotypes [4–9], respectively, have been developed. However, methods as presented in [4–9] have three considerable disadvantages: (D1) they show poor performance in detecting the DAG underlying the observed SK and DK phenotypes; (D2) they have no ability to combine different types of side information, e.g., GI-profile data with SK and DK phenotypes, to enhance the detection quality; and (D3) they cannot make use of prior knowledge in order to enhance the DAG detection quality. Especially, the ability to overcome the disadvantage in (D2) will become more important in the future, since there is a constantly increasing amount of different data types, e.g., SK and DK phenotypes, Pearson correlation-based GI-profile data, and other types of GI-profile data, freely available. Furthermore, the ability to overcome the deficit in (D3), i.e., to incorporate a priori knowledge about the existing results in genomics research into the DAG detection procedure, is also of great significance, since existing functional relationships among genes are increasingly better understood based on a variety of studies that constantly extend the knowledge on the cell machinery and molecular biology. Although exhibiting the abovementioned disadvantages (D1) to (D3), methods as those presented in [4–9] are the most commonly used methods to detect the DAG underlying the measured SK and DK data. Therefore, we propose the Genetic-Interactions-Detector (GENIE) program, that is an approach based on the biological system model of [2] with which it is possible to overcome the abovementioned shortcomings of the most popular methods as those reported in [4–9]. Since the hierarchical relationship classes are mutually dependent, classifying each pair of genes to a specific hierarchical relationship class corresponds to a multi-hypothesis test. Thus, we formulate this multi-hypothesis test as a linear integer optimization program [10–15] in order to find the set of hierarchical relationship classes, best matching the observed SK and DK phenotypes. Based on the detected set of hierarchical relationship classes, the set of edges of the DAG which reflects the interactions among the genes can be computed. Furthermore, we propose the GI-GENIE program where we advance the proposed GENIE program by incorporating GI-profile data, e.g., GI-profile data based on Pearson correlation of the observed SK and DK phenotypes, into the DAG detection procedure. Due to incomplete knowledge about the true dependencies among the very most sets of genes, i.e., the true DAG of a set of genes with respect to a specific cell function is unknown or only partially known for almost all sets of genes irrespectively of the cell function under study, there is a strong interest in the genomics research community in statistically reliable statements about the topology of the DAGs underlying large sets of genes, i.e., for the empirical probability of a pair of genes to interact with each other. Towards this aim, we propose a sequential technique based on the GENIE/GI-GENIE algorithms that yields statistically significant statements about the interactions among genes from a large set of genes under study.

This paper is organized as follows. We first summarize the biological system model of [2] in Section 2, and then, we present in Section 3 the GENIE program for detecting the set of hierarchical relationship classes, which represents a valid DAG and matches the DK measurements best. In Section 4, we extend the GENIE program with GI-profile data (GI-GENIE). In Section 5, we present our scalability approach in order to obtain statistically significant results for large sets of genes. Following Section 5, we present results for simulated data which demonstrate the performance of the GENIE and the GI-GENIE methods in Section 6. Furthermore, in Section 6, we display real data results for the scalability approach described in Section 5. Finally, we summarize in Section 7 the key parts of this paper and give a brief outlook on future work.

## System model

In this section, we provide a mathematical description of a DAG as well as its biological implications. Furthermore, we introduce the common biological terms and provide a compact description of the genetic interaction model of [2] including simple explanations on how to read and interpret a DAG.

### Graph properties of a DAG

According to [16], a graph $\mathcal {A} = \left (\mathrm {V}(\mathcal {A}), \mathrm {E}(\mathcal {A}) \right) $ is well defined by a set of nodes $\mathrm {V}(\mathcal {A}) = \left \{ \mathrm {a}_{1},\mathrm {a}_{2},\ldots,\mathrm {a}_{A} \right \} $ and a set of edges $\mathrm {E}(\mathcal {A})= \left \{ \left \{ \mathrm {a}_{1}, \mathrm {a}_{A},\right \},\left \{ \mathrm {a}_{2}, \mathrm {a}_{A},\right \}, \ldots, \left \{ \mathrm {a}_{A}, \mathrm {a}_{1},\right \} \right \} $ where {a_*i*_,a_*j*_,} for $\mathrm {a}_{i},\mathrm {a}_{j} \in \mathrm {V}(\mathcal {A})$ denotes a directed edge from a_*i*_ to a_*j*_ and cardinality $\left | \mathrm {V}(\mathcal {A}) \right | = A$ denotes the number of elements of set $\mathrm {V}(\mathcal {A})$. The operators V(·) and E(·) applied to graph $\mathcal {A}$ yield the set of nodes $\mathrm {V}(\mathcal {A})$ and the set of edges $\mathrm {E}(\mathcal {A})$ respectively. We mostly address sets $\mathrm {V}(\mathcal {A})$ and $\mathrm {E}(\mathcal {A})$ by $\mathcal {G}_{\mathcal {A}}$ and $\mathcal {E}_{\mathcal {A}}$, respectively, for the sake of notational convenience, i.e., $\mathcal {A} = \left (\mathcal {G}_{\mathcal {A}}, \mathcal {E}_{\mathcal {A}} \right) $. Assume that there is a path P from node $a_{i}\in \mathcal {G}_{\mathcal {A}}$ to node $a_{j} \in \mathcal {G}_{\mathcal {A}}$ in graph $\mathcal {A}$, i.e., a directed connection from node $a_{i}\in \mathcal {G}_{\mathcal {A}}$ to node $a_{j}\in \mathcal {G}_{\mathcal {A}}$. Then, path P is described by the concatenation of nodes being passed through on the way from node $a_{i}\in \mathcal {G}_{\mathcal {A}}$ to node $a_{j}\in \mathcal {G}_{\mathcal {A}}$, i.e., P=*a*_*i*_…*a*_*j*_ and V(P)={*a*_*i*_,…,*a*_*j*_} denotes the set of nodes of path P [16].

The functional dependencies among a set of genes $\mathcal {G} = \left \{ g_{1}, \ldots, g_{G} \right \}$, with $G = \left | \mathcal {G} \right |$ elements, for a given cell process and specie can be characterized by a genetic interaction map (GI map,[17–20]) which is essentially a DAG with a common root node, i.e., the reporter level *R*, [21]. In particular, an arbitrary DAG $\mathcal {D}$ can be described as a graph $\mathcal {D} = \left (\mathcal {G}_{\mathcal {D}},\mathcal {E}_{\mathcal {D}} \right)$ with the set of nodes $\mathcal {G}_{\mathcal {D}} = \left \{ \mathcal {G} \cup R\right \}$ and the set of directed edges $\mathcal {E}_{\mathcal {D}} = \left \{ \left \{ g_{i}, g_{j} \right \},\ldots, \left \{ g_{j}, g_{l} \right \} \right \} $. As the genetic interactions can only be observed through the reporter, all edges are always orientated in such a way that each path parting from any arbitrary gene $g_{i} \in \mathcal {G}$ always terminates in the root node *R* and any gene appears on the path at most once, i.e., there exist no cycles in the graph. Hence, the DAG $\mathcal {D}$ is always connected via its root node *R*. For the sake of notational convenience, in most cases, we write gene *i* when addressing gene *g*_*i*_, [21]. The reporter node *R* is an artificial node, i.e., not a gene, in the concept of a DAG and represents the measured phenotype of the specific cell process under study.

**Fig. 1:**
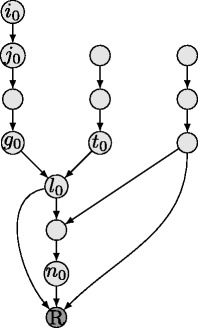
DAG $\mathcal {D}_{0}$ of 13 genes and root node R

To provide a better understanding of the information encoded in a DAG, we state a simple example, which is similar to the one in [21], based on DAG $\mathcal {D}_{0}$ displayed in Fig. 1. In $\mathcal {D}_{0}$, there exists an direct edge from gene *i*_0_ to gene *j*_0_, i.e., $\left \{ i_{0}, j_{0} \right \} \in \mathcal {E}_{\mathcal {D}_{0}}$, which indicates that the activity of gene *i*_0_ controls the activity of gene *j*_0_. Hence, gene *i*_0_ only affects the phenotype via gene *j*_0_ and not directly. We emphasize that in this model, the existence of edge {*i*_0_,*j*_0_} in the DAG only describes the hierarchical functional dependency between genes *i*_0_ and *j*_0_ and not the quantitative effect of gene *i*_0_ on gene *j*_0_.

### Biological interaction model

**Fig. 2:**
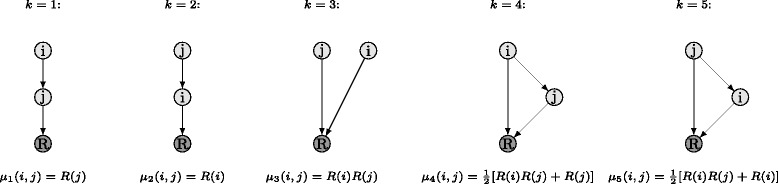
Possible hierarchical relationship classes between two arbitrary genes *i*,*j* of DAG $\mathcal {D}$ according to [2]

Let us denote $R(i) \in \mathbb {R}$ as the phenotype for a single gene $i \in \mathcal {G}$ functionally disabled. In the same way, we define the phenotype for the DK of genes $i,j \in \mathcal {G}$ as $R(i,j) \in \mathbb {R}$. Let the datasets $\mathcal {R}_{i} = \left \{ R(i,1),\ldots,R(i,G)\right \}$ and $\mathcal {R}_{j} = \left \{ R(j,1),\ldots,R(j,G)\right \}$ contain all DK phenotypes involving genes $i,j \in \mathcal {G}$. The GI-profile data *ρ*(*i*,*j*) for genes $i,j \in \mathcal {G}$ can be computed as the Pearson correlation between the samples of the datasets $\mathcal {R}_{i}$ and $\mathcal {R}_{j}$, respectively. We remark that the GI-profile data *ρ*(*i*,*j*) does not have to be separately computed as the Pearson correlation of $\mathcal {R}_{i}$ and $\mathcal {R}_{j}$, respectively. It is commonly extracted from a database where a priori knowledge about the set of genes under study, i.e., $\mathcal {G}$, is stored. Since the gene pairs *i*,*j* and *j*,*i* are identical, it is sufficient to consider only gene pairs $i,j \in \mathcal {G}:j>i$. Throughout this paper, we mostly omit the specification that *j* is greater than *i* for notational convenience. In genomics research, it is a common assumption that if there is an edge between two genes *i*,*j* in DAG $\mathcal {D}$, i.e., there is an interaction between genes *i*,*j* in DAG $\mathcal {D}$, then the GI-profile *ρ*(*i*,*j*) is very likely to be high. Furthermore, according to [2], we assume that each pair of genes *i*,*j* belongs to exactly one out of five hierarchical relationship classes that are characterized in Fig. 2. The hierarchical relationship classes $k \in \mathcal {K} =\left \{1,\ldots,5 \right \} $ are defined according to the model *μ*_*k*_(*i*,*j*) in which the single-knockout phenotypes *R*(*i*) and *R*(*j*) are related with the DK phenotype *R*(*i*,*j*). If the gene pair *i*,*j* belongs to the hierarchical relationship class *k*, then the observed DK phenotype *R*(*i*,*j*) is described by the model *μ*_*k*_(*i*,*j*) provided in Fig. 2. We remark that the five hierarchical dependency graphs in Fig. 2 do not reflect the absolute adjacency relations, but the hierarchical relations between genes *i*,*j* in DAG $\mathcal {D}$. Hence, given that two genes *i*,*j* of DAG $\mathcal {D}$ are in class *k*, we cannot conclude that genes *i*,*j* are directly arranged in DAG $\mathcal {D}$ as displayed by the depiction of class *k* in Fig. 2. This follows from the fact that the description of the hierarchical relationship classes provided in Fig. 2 only contains relative topology information about two genes *i*,*j* in DAG $\mathcal {D}$. In the following and in addition to [2], we provide, for clarity of presentation, a formal description of the hierarchical relationship classes depicted in Fig. 2 using a graph theoretical representation. Assume that there are *I* paths P_*i*,*τ*_,for*τ*∈{1,…,*I*}, from gene *i* to the reporter node *R* in DAG $\mathcal {D}$ and the set $\mathcal {P}_{i}$ containing all such paths is defined as $\mathcal {P}_{i} = \left \{ \mathrm {P}_{i,1},\ldots,\mathrm {P}_{i,I} \right \}$. Furthermore, set $\mathcal {P}_{j} = \left \{ \mathrm {P}_{j,1},\ldots,\mathrm {P}_{j,J} \right \}$ contains all *J* paths from gene *j* to the reporter node *R* in DAG $\mathcal {D}$. Given gene pair *i*,*j* in DAG $\mathcal {D}$, then pair *i*,*j* belongs to the hierarchical relationship class $k \in \mathcal {K}$ if and only if condition C_*k*_ as defined below is satisfied:

1a $$\begin{array}{*{20}l} \mathrm{C}_{1}: &  \\ & \forall \ \mathrm{P}_{i,\tau} \in \mathcal{P}_{i} : j \in \mathrm{V}(\mathrm{P}_{i,\tau})  \\ &  \end{array} $$

1b $$\begin{array}{*{20}l} \mathrm{C}_{2}: &  \\ & \forall \ \mathrm{P}_{j,\tau} \in \mathcal{P}_{j} : i \in \mathrm{V}(\mathrm{P}_{j,\tau})  \\ &  \end{array} $$

1c $$\begin{array}{*{20}l} \mathrm{C}_{3}: &  \\ & \left(\forall \ \mathrm{P}_{i,\tau} \in \mathcal{P}_{i} : j \notin \mathrm{V}(\mathrm{P}_{i,\tau}) \right) \bigwedge  \\ & \left(\forall \ \mathrm{P}_{j,\tilde{\tau}} \in \mathcal{P}_{j} : i \notin \mathrm{V}(\mathrm{P}_{j,\tilde{\tau}}) \right) \\ &  \end{array} $$

1d $$\begin{array}{*{20}l} \mathrm{C}_{4}: & \\ & \left(\exists \ \mathrm{P}_{i,\tau} \in \mathcal{P}_i: j \notin \mathrm{V}(\mathrm{P}_{i,\tau}) \right) \bigwedge  \\ & \left(\exists \ \mathrm{P}_{i,\tau} \in \mathcal{P}_{i}, \mathrm{P}_{j,\tilde{\tau}} \in \mathcal{P}_{j} : \mathrm{V}(\mathrm{P}_{j,\tilde{\tau}}) \subset \mathrm{V}(\mathrm{P}_{i,\tau}) \right)  \\ &  \end{array} $$

1e $$\begin{array}{*{20}l} \mathrm{C}_{5}:&  \\ & \left(\exists \ \mathrm{P}_{j,\tilde{\tau}} \in \mathcal{P}_j: i \notin \mathrm{V}(\mathrm{P}_{j,\tilde{\tau}}) \right) \bigwedge  \\ & \left(\exists \ \mathrm{P}_{i,\tau} \in \mathcal{P}_{i}, \mathrm{P}_{j,\tilde{\tau}} \in \mathcal{P}_{j} : \mathrm{V}(\mathrm{P}_{i,\tau}) \subset \mathrm{V}(\mathrm{P}_{j,\tilde{\tau}}) \right)  \\ & \end{array} $$

As stated in condition C_1_ in (1a), two genes *i*,*j* in DAG $\mathcal {D}$ belong to the hierarchical relationship class *k*=1, if all paths from gene *i* to the reporter node *R* pass through gene *j*. Hence, gene *j* is always an element of the set of nodes of each path $\mathrm {P}_{i,\tau } \in \mathcal {P}_{i}$ from gene *i* to the reporter node *R*, i.e., *j*∈V(P_*i*,*τ*_) for all paths P_*i*,*τ*_ from gene *i* to the reporter node *R*. With the same line of argument as used in (1a), two genes *i*,*j* in DAG $\mathcal {D}$ belong to the hierarchical relationship class *k*=2 if condition C_2_ in (1b) is satisfied. Two genes *i*,*j* in DAG $\mathcal {D}$ belong to the hierarchical relationship class *k*=3 and are considered to be independent from each other if condition C_3_ in (1c) is satisfied which states that there is no path P_*i*,*τ*_ from gene *i* to the reporter node *R* that passes through gene *j* as well as there is no path $\mathrm {P}_{j,\tilde {\tau }}$ from gene *j* to the reporter node *R* that passes through gene *i*. As stated in (1d), two genes *i*,*j* in DAG $\mathcal {D}$ belong to the hierarchical relationship class *k*=4 if there is at least one path P_*i*,*τ*_ from gene *i* to the reporter node *R* which does not pass through gene *j* as well as for all paths $\mathrm {P}_{j,\tilde {\tau }} \in \mathcal {P}_{j}$, there is always a path $\mathrm {P}_{i,\tau } \in \mathcal {P}_{i}$ that is a super-path of the respective $\mathrm {P}_{j,\tilde {\tau }} \in \mathcal {P}_{j}$. With the same line of argument as used in (1d), two genes *i*,*j* in DAG $\mathcal {D}$ belong to the hierarchical relationship class *k*=5 if condition C_5_ in (1e) is satisfied.

### Class coupling—example

To illustrate this, let us consider the example DAG $\mathcal {D}_{0}$ of Fig. 1. All paths from gene *i*_0_ to node *R* pass through gene *j*_0_, i.e., they are in a linear pathway with gene *i*_0_ upwards of gene *j*_0_. Thus, the pair of genes *i*_0_,*j*_0_ belongs to class *k*=1. Note that with the same line of argument, we conclude that also genes *i*_0_ and *l*_0_ belong to relationship class *k*=1. Since all paths from gene *i*_0_ to the reporter level *R* do not pass through gene *t*_0_ and all paths from gene *t*_0_ to the reporter level do not pass through gene *i*_0_, genes *i*_0_ and *t*_0_ belong to the hierarchical relationship class *k*=3 as given in Fig. 2, which states that genes *i*_0_ and *t*_0_ are independent of each other and the DK phenotype amounts to *R*(*i*_0_,*t*_0_)=*μ*_3_(*i*_0_,*t*_0_). Finally, let us investigate the hierarchical relation between genes *t*_0_ and *n*_0_ in DAG $\mathcal {D}_{0}$. Obviously, gene *t*_0_ has (at least) one path to node *R* which does not pass through gene *n*_0_, i.e., genes only having paths to *R* that do not pass through gene *n*_0_ do not affect the activity of gene *n*_0_. Since there is (at least) one other path from gene *t*_0_ to *R* passing through gene *n*_0_, we can reason that genes *t*_0_ and *n*_0_ belong to class *k*=4. Generally, there are strong implications among the hierarchical relationship classes of [2], i.e., if some pairs belong to a specific class, then this has strong implications for all other pairs. Let us consider the case that DAG $\mathcal {D}_{0}$ was not known and only the hierarchical relationship classes for genes *i*_0_ and *j*_0_, i.e., genes *i*_0_ and *j*_0_ belong to class *k*=1, as well as the hierarchical relationship class for genes *i*_0_ and *g*_0_, i.e., genes *i*_0_ and *g*_0_ belong to class *k*=1, were available. By definition of the hierarchical dependency graphs in Fig. 2 and the assumptions that genes *i*_0_ and *j*_0_ belong to class *k*=1 as well as that genes *i*_0_ and *g*_0_ belong to class *k*=1, we conclude that all paths from gene *i*_0_ to *R* pass through genes *j*_0_ and *g*_0_. Thus, either all paths from gene *g*_0_ to *R* pass through gene *j*_0_ or all paths from gene *j*_0_ to *R* pass through gene *g*_0_. Consequently, genes *j*_0_ and *g*_0_ either belong to the hierarchical relationship class *k*=1, or *k*=2.

As we have emphazised by the example above, generally, if the hierarchical relationship class is known for two arbitrary genes *i*,*j* as well as for another pair $i,l \in \mathcal {G}: l>i$, then this has strong logical implications on the hierarchical relationship classes genes $j,l \in \mathcal {G}:l\;>j$ can belong to. Since we can interpret the classification of the pairs of genes *i*,*j*, based on their observed SK and DK phenotypes *R*(*i*),*R*(*j*) and *R*(*i*,*j*), respectively, to exactly one out of the five hierarchical relationship classes as a coupled multi-hypothesis test, we address this problem in Section 3 by a linear integer optimization program. The proposed linear integer optimization program identifies the most consistent set of hierarchical relationship classes, i.e., the set of hierarchical relationship classes that represents a valid DAG and matches best the DK measurements with respect to the logical coupling between the classes. Furthermore, in Section 4, we extend the GENIE program proposed in Section 3 by incorporating GI-profile data in order to jointly detect the most consistent set of hierarchical relationship classes and the corresponding DAG topology.

## GENIE algorithm

In this section, we formulate the problem of classifying the gene pairs *i*,*j* into the classes of hierarchical relationships based on the observed SK and DK phenotype values as a linear integer optimization program. Furthermore, we translate the logical implications among the hierarchical relationship classes into constraints that ensure that the detected set of hierarchical relationship classes represents a valid graph. That is, the detected set of hierarchical relationship classes represents a graph which is a DAG as defined in Section 2. Finally, we propose a policy to derive an estimate $\hat {\mathcal {E}}_{\mathcal {D}}$ of the true set of edges $\mathcal {E}_{\mathcal {D}}$ of DAG $\mathcal {D}$ based on the detected set of hierarchical relationship classes.

### Hierarchical relationship class detection

In order to quantify the mismatch between the measured DK phenotypes *R*(*i*,*j*) and the phenotype model *μ*_*k*_(*i*,*j*) of class $k \in \mathcal {K}$ according to Fig. 2, under the hypothesis that the gene pairs *i*,*j* belong to class *k* given their respective SK values, we propose a simple quadratic score [2, 21], as given in Eq. (2)

2 $$\begin{array}{*{20}l} s_{k}(i,j) = \left(R(i,j) - \mu_{k}(i,j) \right)^{2}, \quad k \in \mathcal{K}  \\ \forall i,j:\in \mathcal{G}: j>i  \end{array} $$

Let us define the following class-selection variables^1^

3 $$\begin{array}{*{20}l} \alpha_{k}(i,j) = \left\{\begin{array}{ll} 1 & \text{if } i,j \text{are in class} {k} \\ 0 & \text{else} \end{array}\right.  \\ k \in \mathcal{K}, \quad \forall i,j:\in \mathcal{G}: j>i  \end{array} $$

We remark that every DAG $\mathcal {D}$ can be represented by a set of hierarchical relationship classes which directly corresponds to a set of class-selection variables $A^{\mathcal {D}} =\bigcup \limits _{\forall i,j \in \mathcal {G}: j>i} \left \{ \alpha _{1}^{\mathcal {D}}(i,j),\ldots, \alpha _{5}^{\mathcal {D}}(i,j) \right \} $. The GENIE algorithm of classifying the gene pairs *i*,*j* into the set of hierarchical relationship classes that represents a valid DAG and matches the observed SK and DK phenotypes best can be formulated as

4a $$\begin{array}{*{20}l} \mathrm{O}_{\text{GENIE}}: &  \\ \min_{\left\{ \alpha_{k}(i,j) \right\}} \; & \quad \sum\limits_{i=1}^{G} \sum\limits_{j=i+1}^{G} \left(\sum\limits_{k=1}^{\left| \mathcal{K} \right|} s_{k}(i,j)\alpha_{k}(i,j) \right)  \end{array} $$

4b $$\begin{array}{*{20}l} \text{s. t.} \; & \quad \alpha_{k}(i,j) \in \left\{ 0,1 \right\} \ \forall k \in \mathcal{K}, \\ & \quad \forall i,j \in \mathcal{G}: j>i  \end{array} $$

4c $$\begin{array}{*{20}l} & \quad \sum\limits_{k=1}^{\left| \mathcal{K} \right|} \alpha_{k}(i,j) = 1,  \\ & \quad \forall i,j \in \mathcal{G}: j>i  \end{array} $$

4d $$\begin{array}{*{20}l} & \quad \mathcal{L} \Longrightarrow \text{additional topology}  \\ & \quad \quad \qquad \text{constraints}  \end{array} $$

where $A^{\mathrm {O}_{\text {GENIE}}} = \bigcup \limits _{\forall i,j \in \mathcal {G}: j>i }\left \{ \alpha _{1}^{\mathrm {O}_{\text {GENIE}}}(i,j),\ldots, \alpha _{5}^{\mathrm {O}_{\text {GENIE}}}(i,j) \right \}$ denotes the solution of program O_GENIE_ in (4) and the set of best matching selection variables $A^{\mathrm {O}_{\text {GENIE}}}$ corresponds to the most consistent pattern of hierarchical relationship classes. Problem O_GENIE_ in (4) is a linear integer program which can be solved efficiently by BB methods [22–29]. The objective of problem O_GENIE_ is to minimize the overall mismatch in classifying each gene pair *i*,*j* to one out of five hierarchical relationship classes. The constraints in (4b) reflect the binary nature of the class-selection variables *α*_*k*_(*i*,*j*), $\forall k \in \mathcal {K}$, while (4c) represents a multiple choice constraint to enforce that the gene pairs *i*,*j* are only classified to one out of the five hierarchical relationship classes. The set $\mathcal {L}$ in (4d) is comprised of additional constraints to ensure that the detected set of selection variables $A^{\mathrm {O}_{\text {GENIE}}}$ always represents a valid graph, i.e., a DAG. In the following, we exemplarily derive topology constraints in set $\mathcal {L}$. In order to identify the numerous logical dependencies among the class-selection variables $\alpha _{k}(i,j), k \in \mathcal {K}$ for all $i,j \in \mathcal {G}:j>i$, we proceed in the following way. We first fix the assumption that genes *i*,*j* belong to class *k*=1, i.e., *α*_1_(*i*,*j*)=1. Further, we assume that genes $i,l \in \mathcal {G}:l>i$ belong to class *k* ^′^, i.e., $\alpha _{k^{\prime }}(i,l)=1$. Then, we derive the set of classes $\mathcal {K}^{\prime \prime }$ that genes $j,l \in \mathcal {G}:l>j$ can belong to under the assumptions made. In the following, we have formulated the logical dependencies among the selection variables for *α*_1_(*i*,*j*)=1, i.e., the case that gene *i* is linearly upstream of gene *j*, as linear integer inequalities defined in constraints (5a)–(5e) and summarize them as set $\mathcal {L}_{1}$

5a $$\begin{array}{*{20}l} \mathcal{L}_{1} = \left\{{\vphantom{\frac{0}{0}}}\right.&  \\ & \alpha_{1}(j,l) +\alpha_{2}(j,l) \geq \alpha_{1}(i,j)+ \alpha_{1}(i,l) -1  \end{array} $$

5b $$\begin{array}{*{20}l} & \alpha_{2}(j,l) \geq \alpha_{1}(i,j)+ \alpha_{2}(i,l) -1  \end{array} $$

5c $$\begin{array}{*{20}l} & \alpha_{2}(j,l) + \alpha_{3}(j,l) + \alpha_{5}(j,l) \geq  \\ & \alpha_{1}(i,j)+ \alpha_{3}(i,l) -1  \end{array} $$

5d $$\begin{array}{*{20}l} & \alpha_{2}(j,l) + \alpha_{4}(j,l) \geq \alpha_{1}(i,j)+ \alpha_{4}(i,l) -1  \end{array} $$

5e $$\begin{array}{*{20}l} & \alpha_{5}(j,l) + \alpha_{2}(j,l) \geq \alpha_{1}(i,j)+ \alpha_{5}(i,l) -1  \end{array} $$

$$\begin{array}{*{20}l} & \left.{\vphantom{\frac{0}{0}}}\right\} \forall i,j,l \in \mathcal{G}: l >j>i  \end{array} $$

where constraints (5a)–(5e) are linear after the continuous relaxation of the selection variables *α*_*k*_(*i*,*j*). To explain the origin and the functionality of the constraints in $\mathcal {L}_{1}$, let us further define a sub-genetic interaction map (SMAP) $\mathcal {S}$, [21], as given in Fig. 3 according to the following definition where we adopt the graph notation of [16]:

**Fig. 3:**
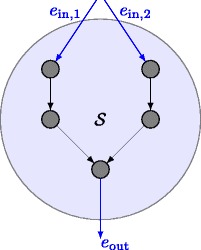
Example SMAP $\mathcal {S}$

#### **Definition 1**

Given a non-empty set of edges $\mathcal {E}_{\text {in}}$ and a non-empty set of edges $\mathcal {E}_{\text {out}}$, graph $\mathcal {S} = \left (\mathcal {G}_{\mathcal {S}}, \mathcal {E}_{\mathcal {S}} \right)$, with set of nodes $\mathcal {G}_{\mathcal {S}}$ and set of edges $\mathcal {E}_{\mathcal {S}}$, is a SMAP if the following conditions are fulfilled: (*i*) the graph $\mathcal {S}$ is acyclic and directed and (*i* *i*) there are $\exists e_{\text {in}} \in \mathcal {E}_{\text {in}} ~{and}~ e_{\text {out}} \in \mathcal {E}_{\text {out}}$ such that each path P through graph $\mathcal {S}$ incides $\mathcal {S}$ via egde *e*_in_ and leaves graph $\mathcal {S}$ via edge *e*_out_.

**Fig. 4:**
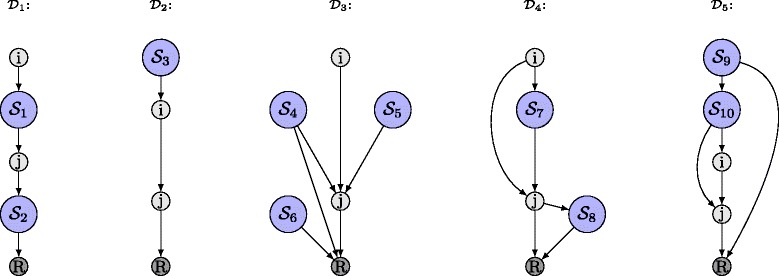
Schematically reduced DAGs $\mathcal {D}_{1}$ to $\mathcal {D}_{5}$ corresponding to Eqs. (5a)–(5e), respectively

DAG $\mathcal {D}_{1}$, as displayed in Fig. 4, consists of genes *i*,*j* and SMAPs $\mathcal {S}_{1}$ and $\mathcal {S}_{2}$. Obviously, genes *i*,*j* belong to class *k*=1, i.e., *α*_1_(*i*,*j*)=1. Furthermore, all genes $l \in \mathcal {G}_{\mathcal {D}_{1}} \setminus \{R\}:l>j>i$ for which *α*_1_(*i*,*l*)=1 must be either located in SMAP $\mathcal {S}_{1}$ or $\mathcal {S}_{2}$. Thus, it follows from DAG $\mathcal {D}_{1}$ in Fig. 4 that the gene pair *j*,*l* is either in hierarchical relationship class *k*=1 or *k*=2, i.e., *α*_1_(*j*,*l*)=1 or *α*_2_(*j*,*l*)=1.

This logical implication is directly reflected by constraint (5a). Given *α*_1_(*i*,*j*)=1 and *α*_1_(*i*,*l*)=1, the right-hand side (RHS) of (5a) amounts to 1. In this case also, the left-hand side (LHS) of (5a) becomes 1 to fulfill the inequality (5a). Thus, either *α*_1_(*j*,*l*)=1 or *α*_2_(*j*,*l*)=1. Reversely, assume that *α*_1_(*i*,*j*)=1 and *α*_1_(*i*,*l*)=1 does not hold, and then, the RHS of (5a) is less than 1, i.e., 0 or −1, while the LHS of (5a) is always greater than 0. Hence, constraint (5a) is fulfilled irrespectively of the choice of *α*_*k*_(*j*,*l*), i.e., constraint (5a) enforces no logical implications.

Similarly for DAG $\mathcal {D}_{2}$ in Fig. 4, it is obvious that genes *i*,*j* belong to the hierarchical relationship class *k*=1, i.e., *α*_1_(*i*,*j*)=1. All genes $l \in \mathcal {G}_{\mathcal {D}_{2}} \setminus \{R\}: l>j>i$ which are in a linear pathway upstream of gene *i*, i.e., *α*_2_(*i*,*l*)=1, must be located in SMAP $\mathcal {S}_{3}$. Hence, it directly follows from DAG $\mathcal {D}_{2}$ that also, gene *l* must be in a linear pathway upstream of gene *j*, i.e., *α*_2_(*j*,*l*)=1. This logical implication is compactly represented in constraint (5b). Under the assumption that *α*_1_(*i*,*j*)=1 and *α*_2_(*i*,*l*)=1, the RHS of (5b) amounts to 1 enforcing *α*_2_(*j*,*l*)=1, so that the LHS of (5b) equals the RHS and the inequality in (5b) is fulfilled. Reversely, assume that *α*_2_(*i*,*l*)=0, then the RHS of (5b) is less than 1 and hence the LHS of (5b) is always bigger than or equal to the RHS irrespectively of the choice of *α*_*k*_(*j*,*l*), i.e., constraint (5a) enforces no logical implications. We can proceed in the same fashion to explain constraints (5c)–(5e) based on DAGs $\mathcal {D}_{3}$ to $\mathcal {D}_{5}$ as given in Fig. 4, respectively. Note that the DAGs $\mathcal {D}_{1}$ to $\mathcal {D}_{5}$ are sufficient illustrations of Eqs. (5a)– (5e) in order to derive all logical implications for the case that genes *i*,*j* are in class 1, i.e., *α*_1_(*i*,*j*)=1. In the case of DAG $\mathcal {D}_{1}$, for instance, there can be other DAGs than DAG $\mathcal {D}_{1}$ indeed where genes *i*,*j* are in class 1 and genes *i*,*l* are in class 1, i.e. *α*_1_(*i*,*j*)=1 and *α*_1_(*i*,*l*)=1. However, DAG $\mathcal {D}_{1}$ contains all the necessary information in order to derive the logical implications for gene pair *j*,*l*, given that *α*_1_(*i*,*j*)=1 and *α*_1_(*i*,*l*)=1. The same holds for DAGs $\mathcal {D}_{2}$ to $\mathcal {D}_{5}$. Furthermore, with the same line of argument, we can derive the sets $\mathcal {L}_{k}$ for $k \in \mathcal {K} \setminus 1$ which reflect the logical implications among the selection variables under the assumptions that *α*_*k*_(*i*,*j*)=1 for $k \in \mathcal {K} \setminus 1$. However, due to space limitations, we omit the derivation of the full set of logical implications at this point and refer the interested reader to [30] where we will provide the full set of topology constraints $\mathcal {L}$ as well as further supplementary material. The full set of topology constraints $\mathcal {L}$ in (4d) can be computed as

6 $$\begin{array}{*{20}l} \mathcal{L} = \bigcup\limits_{k=1}^{\left| \mathcal{K} \right|} \left\{ \mathcal{L}_{k} \right\}. \end{array} $$

Finally, a considerable advantage of the presented algorithm is its ability to incorporate prior knowledge into the classification of the gene pairs *i*,*j* to the most consistent hierarchical relationship classes. Assume that it is known from existing experimental results that two specific genes $i_{0},j_{0} \in \mathcal {G} :j_{0}>i_{0}$ do not interact with each other. Then, we can easily incorporate this prior knowledge into program O_GENIE_ in (4) by adding Eq. (7) as defined below

7 $$\begin{array}{*{20}l} \alpha_{3}(i_{0},j_{0}) = 1  \end{array} $$

as a topology constraint to program O_GENIE_. This property is very valuable since it allows the GENIE algorithm to take advantage of existing results in genetic interaction research to improve the reliability of the classification.

### Edge computation

**Fig. 5:**
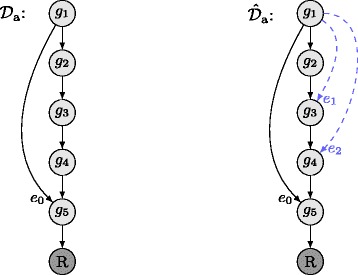
Left: Original DAG $\mathcal {D}_{a}$ with corresponding set of hierarchical relationship classes $A^{\mathcal {D}_{a}}$. Right: Reconstruction $\hat {\mathcal {D}}_{a}$ of DAG $\mathcal {D}_{a}$ based on $A^{\mathcal {D}_{a}}$

Based on the detected set of selection variables $A^{\mathrm {O}_{\text {GENIE}}}$ which corresponds to the most consistent pattern of hierarchical relationship classes given the observed SK and DK phenotypes, an estimate $\mathcal {E}_{\text {GENIE}}$ of the true set of edges $\mathcal {E}_{\mathcal {D}}$ of DAG $\mathcal {D}$ can be computed. It can be theoretically proven that the representation of an arbitrary DAG $\mathcal {D}$ by its corresponding set of hierarchical relationship classes is not unique. $\mathrm {A}^{\mathcal {D}}$ the set of selection variables which directly corresponds to the hierarchical relationship class pattern of DAG $\mathcal {D}$ represents not only the true DAG $\mathcal {D}$ but also a set of similar DAGs which have minorly different sets of edges compared to the true DAG $\mathcal {D}$. Assume we are only given that $\alpha _{4}^{\mathcal {D}}(i,j)=1$ for two arbitrary genes *i*,*j* of DAG $\mathcal {D}$, then we suffer an information loss on the number of paths from gene *i* to the reporter node *R* which are independent of gene *j*. Similarly, given that $\alpha _{5}^{\mathcal {D}}(i,j)=1$ for two arbitrary genes $i,j \in \mathcal {G}:j>i$ of DAG $\mathcal {D}$, we suffer an information loss on the number of paths from gene *j* to the reporter node *R* which are independent of gene *i*. Hence, this information loss yields ambiguities in computing the set of edges $\mathcal {E}_{\mathcal {D}}$ of DAG $\mathcal {D}$ based on the $\mathrm {A}^{\mathcal {D}}$. In order to clarify the origin of the ambiguities further, let us turn to a simple example. Given DAG $\mathcal {D}_{a} = \left \{\mathcal {G}_{\mathcal {D}_{a}},\mathcal {E}_{\mathcal {D}_{a}} \right \}$ as displayed on the LHS of Fig. 5 and the corresponding set of hierarchical relationship classes represented by the corresponding set of selection variables $\mathrm {A}^{\mathcal {D}_{a}}$. Note that $\alpha _{4}^{\mathcal {D}_{a}}(1,2) = \alpha _{4}^{\mathcal {D}_{a}}(1,3) = \alpha _{4}^{\mathcal {D}_{a}}(1,4) = 1$ due to edge $e_{0} \in \mathcal {E}_{\mathcal {D}_{a}}$ and $\alpha _{1}^{\mathcal {D}_{a}}(1,5) = 1$. Now, assume that we want to compute the topology of DAG $\mathcal {D}_{a}$, i.e., the set of edges $\mathcal {E}_{\mathcal {D}_{a}}$, based on $\mathrm {A}^{\mathcal {D}_{a}}$. DAG $\hat {\mathcal {D}}_{a}$ displayed on the RHS of Fig. 5 shows the estimated topology of DAG $\mathcal {D}_{a}$ based on $\mathrm {A}^{\mathcal {D}_{a}}$. It can be shown that the black edges of DAG $\hat {\mathcal {D}}_{a}$ are necessary such that DAG $\hat {\mathcal {D}}_{a}$ is represented by $\mathrm {A}^{\mathcal {D}_{a}}$. Edges *e*_1_ and *e*_2_ in DAG $\hat {\mathcal {D}}_{a}$ are optional in a sense that their existence has no effect on set $\mathrm {A}^{\mathcal {D}_{a}}$. Edges *e*_1_ and *e*_2_ create two paths from gene *g*_1_ to the reporter node *R* which are independent of gene *g*_2_ and gene *g*_3_, respectively. However, due to edge *e*_0_, gene *g*_1_ already has a path to the reporter node *R* which is independent of genes *g*_2_ and *g*_3_. Since $\alpha _{4}^{\mathcal {D}_{a}}(1,2) = \alpha _{4}^{\mathcal {D}_{a}}(1,3) = \alpha _{4}^{\mathcal {D}_{a}}(1,4) = 1$ do not contain information on the number of paths from gene *g*_1_ to *R* that are independent of *g*_2_, *g*_3_ and *g*_4_, edges *e*_1_ and *e*_2_ do not affect the pattern of hierarchical relationship classes representing DAG $\mathcal {D}_{a}$, i.e., $\mathrm {A}^{\mathcal {D}_{a}}$, and hence, this yields ambiguities in computing the topology of DAG $\mathcal {D}_{a}$ based on its corresponding set of selection variables $\mathrm {A}^{\mathcal {D}_{a}}$. Since it is a common assumption in genomics research that GI maps, i.e., DAGs, are not overly dense but rather sparse, we propose a policy which computes the sparsest DAG topology based on the detected pattern of hierarchical relationship classes. Given the detected pattern of hierarchical relationship classes of a DAG $\mathcal {D}$, i.e., $\hat {\mathrm {A}}^{\mathcal {D}} =\bigcup \limits _{\forall i,j \in \mathcal {G}: j>i} \left \{ \hat {\alpha }_{1}^{\mathcal {D}}(i,j),\ldots, \hat {\alpha }_{5}^{\mathcal {D}}(i,j) \right \} $, we compute an estimate $\hat {\mathcal {E}}_{\mathcal {D}}$ of the true topology set $\mathcal {E}_{\mathcal {D}}$ of DAG $\mathcal {D}$ according to the policy depicted in Table 1 where we make use of the symmetry properties $\hat {\alpha }_{1}^{\mathcal {D}}(i,j) = \hat {\alpha }_{2}^{\mathcal {D}}(j,i)$, $\hat {\alpha }_{2}^{\mathcal {D}}(i,j) = \hat {\alpha }_{1}^{\mathcal {D}}(j,i)$, $\hat {\alpha }_{3}^{\mathcal {D}}(i,j) = \hat {\alpha }_{3}^{\mathcal {D}}(j,i)$, $\hat {\alpha }_{4}^{\mathcal {D}}(i,j) = \hat {\alpha }_{5}^{\mathcal {D}}(j,i)$, and $\hat {\alpha }_{5}^{\mathcal {D}}(i,j) = \hat {\alpha }_{4}^{\mathcal {D}}(j,i)$. Note that we redundantly expand the set of detected class-selection variables $\hat {\alpha }_{k}(i,j)$ from all pairs $i,j \in \mathcal {G}:j>i$ to all pairs $i,j \in \mathcal {G}$ in order to obtain a compact formulation of the mutually exclusive conditions E_1_ to E_4_ as stated in Table 1.

**Table 1:** Proposed sparse edge detection policy

Assume that either condition E_1_ or condition E_2_ is fulfilled, then we conclude that there is an edge from gene *i* to gene *j* in DAG $\mathcal {D}$. Given that either condition E_3_ or condition E_4_ is fulfilled, we conclude that there exists an edge from gene *j* to gene *i* in DAG $\mathcal {D}$. We remark that there cannot be an edge between two genes *i*,*j* if they are independent of each other, i.e., $\hat {\alpha }_{3}^{\mathcal {D}}(i,j)=1$.

**Fig. 6:**
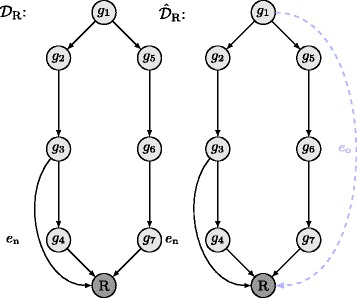
Example DAG $\mathcal {D}_{\mathrm {R}}$ to elucidate the functionality of the RHS of condition E_*R*_ of Table 2

As described by E_1_, there is an edge from gene *i* to gene *j* in DAG $\mathcal {D}$, if gene *i* is linearly upstream of gene *j*, i.e., $\hat {\alpha }_{1}^{\mathcal {D}}(i,j)=1$, and there is no gene *l* in DAG $\mathcal {D}$ that is linearly downstream of gene *i*, i.e., $\hat {\alpha }_{1}^{\mathcal {D}}(i,l)=1$, and linearly upstream of gene *j*, i.e., $\hat {\alpha }_{2}^{\mathcal {D}}(j,l)=1$. According to condition E_2_, there is an edge from gene *i* to gene *j* in DAG $\mathcal {D}$, if gene *i* is upstream of gene *j* with at least one path from gene *i* to *R* which is independent of gene *j*, and furthermore, there is no gene *l* in DAG $\mathcal {D}$ that is either linearly downstream of gene *i* or downstream of gene *i* with gene *i* having at least one path to *R* that is independent of *l* and neither gene *l* is linearly upstream of gene *j* nor gene *l* is upstream of gene *j* with an independent path to *R*. In order to elucidate the effect of condition E_2_ onto the edge computation, we briefly turn to DAG $\hat {\mathcal {D}}_{a}$ in Fig. 5. Condition E_2_ ensures that the optional edges *e*_1_ and *e*_2_ are not detected but only the necessary edges displayed in black color. We remark that conditions E_3_ and E_4_ can be elucidated by the same line of argument as used for conditions E_1_ and E_2_, but due to space limitations, we omit a detailed explanation at this point. Finally, we propose a condition from which all reporter node edges, i.e, all edges that connect gene $i \in \mathcal {G}$ with reporter node *R* in DAG $\mathcal {D}$, can be computed. Based on the detected set of hierarchical relationship classes, i.e., $\hat {\mathrm {A}}^{\mathcal {D}}$, we follow our policy of computing the necessary edges only. For clarity of presentation and notational compactness, we define set $\mathcal {M}_{i}$ as

8 $$\begin{array}{*{20}l} \mathcal{M}_{i} = \left\{ l \in \mathcal{G} \setminus i | \ \hat{\alpha}_{4}^{\mathcal{D}}(i,l) = 1 \right\}  \\ i=1,\ldots,G \end{array} $$

containing all genes $l \in \mathcal {G}$ which are in class *k*=4 with gene $i \in \mathcal {G}$, i.e., $\hat {\alpha }_{4}^{\mathcal {D}}(i,l) = 1$. Furthermore, we define set $\mathcal {M}_{i}^{\prime }$ as

9 $$\begin{array}{*{20}l} \mathcal{M}_{i}^{\prime} = \left\{ l \in \mathcal{M}_{i} | \ \exists \tilde{l} \in \mathcal{M}_{i} \setminus l: \hat{\alpha}_{3}^{\mathcal{D}}(l,\tilde{l}) = 1 \right\}  \\ \quad i=1,\ldots,G  \end{array} $$

which contains all genes *l* of set $\mathcal {M}_{i}$ that are independent of at least one other gene of set $\mathcal {M}_{i}$. Based on sets $\mathcal {M}_{i}$ and $\mathcal {M}_{i}^{\prime }$, we formulate condition E_*R*_ as stated in Table 2. We conclude that there is an edge from gene *i* to reporter node *R* in DAG $\mathcal {D}$, if condition E_*R*_ is fulfilled. Given that gene *i* is linearly upstream of at least a single gene *l*, i.e., $\hat {\alpha }_{1}^{\mathcal {D}}(i,l) = 1$, there cannot exist an edge from gene *i* to reporter node *R* in DAG $\mathcal {D}$, since all paths from gene *i* to *R* pass through at least one other gene *l*. Conversely, if there is no such gene *l* that $\hat {\alpha }_{1}^{\mathcal {D}}(i,l) = 1$, then the LHS of E_*R*_ as given in Table 2 is fulfilled. The RHS of E_*R*_ accounts for our policy of detecting sparse DAGs only and is fulfilled if either $\mathcal {M}_{i}$, $\mathcal {M}_{i}^{\prime }$, or $\mathcal {M}_{i}$ and $\mathcal {M}_{i}^{\prime }$ are empty. Note that given $\mathcal {M}_{i} = \emptyset $, it follows that $\mathcal {M}_{i}^{\prime } = \emptyset $ as well, whereas the opposite is not true. In order to explain the effect of the RHS of condition E_*R*_ in an intuitive manner, let us turn to DAG $\mathcal {D}_{\mathrm {R}}$ as displayed in Fig. 6. Assume that we are given the pattern of hierarchical relationship classes that corresponds to DAG $\mathcal {D}_{\mathrm {R}}$, i.e., $A^{\mathcal {D}_{\mathrm {R}}}$, and we want to compute all reporter node edges based on $A^{\mathcal {D}_{\mathrm {R}}}$, i.e., all edges that directly connect a gene in DAG $\mathcal {D}_{\mathrm {R}}$ with the reporter node *R*. Note that $\alpha _{1}^{\mathcal {D}_{\mathrm {R}}}(2,3) = 1$, $\alpha _{5}^{\mathcal {D}_{\mathrm {R}}}(2,4) = 1$, $\alpha _{4}^{\mathcal {D}_{\mathrm {R}}}(3,4) = 1$, and $\alpha _{4}^{\mathcal {D}_{\mathrm {R}}}(1,l) = 1 \ \forall l\in \mathcal {G}_{\mathcal {D}_{R}} \setminus \left \{ g_{1}, R\right \}$. It can be shown that gene *g*_3_ fulfills the LHS of E_*R*_, i.e., there is no gene which is linearly downstream of *g*_3_, and furthermore, $\mathcal {M}_{3} = \left \{ g_{4} \right \}$ and $\mathcal {M}_{3}^{\prime } = \emptyset $. Hence, condition E_*R*_ is fulfilled and edge *e*_*n*_ connecting *g*_3_ and *R* is computed in DAG $\hat {\mathcal {D}}_{\mathrm {R}}$ that is the reconstruction of DAG $\mathcal {D}_{\mathrm {R}}$ based on $A^{\mathcal {D}_{\mathrm {R}}}$. Furthermore, for set $A^{\mathcal {D}_{\mathrm {R}}}$, the edge *e*_*n*_ in the reconstructed DAG $\hat {\mathcal {D}}_{\mathrm {R}}$ is necessary, since $\alpha _{1}^{\mathcal {D}_{\mathrm {R}}}(2,3) = 1$, $\alpha _{5}^{\mathcal {D}_{\mathrm {R}}}(2,4) = 1$, and $\alpha _{4}^{\mathcal {D}_{\mathrm {R}}}(3,4) = 1$. In contrast to *e*_*n*_, edge *e*_*o*_ is not necessary for $A^{\mathcal {D}_{\mathrm {R}}}$ to represent $\hat {\mathcal {D}}_{\mathrm {R}}$, since $\alpha _{4}^{\mathcal {D}_{\mathrm {R}}}(1,l) = 1 \ \forall l\in \mathcal {G}_{\mathcal {D}_{R}} \setminus \left \{ g_{1}, R\right \}$ irrespectively of edge *e*_*o*_. Hence, *e*_*o*_ is not detected, since $\mathcal {M}_{1} \neq \emptyset $ and $\mathcal {M}_{1}^{\prime } \neq \emptyset $.

**Table 2:** Proposed reporter node edge detection policy

We obtain an estimate $\mathcal {E}_{\text {GENIE}}$ of the true set of edges $\mathcal {E}_{\mathcal {D}}$ of DAG $\mathcal {D}$ by setting $\hat {\mathrm {A}}^{\mathcal {D}} = A^{\mathrm {O}_{\text {GENIE}}}$ and evaluating conditions E_1_ to E_4_ and condition E_*R*_ as stated in Tables 1 and 2, respectively.

## GI-GENIE algorithm

In this section, we present the proposed GI-GENIE algorithm which jointly formulates the gene pair classification and the corresponding DAG topology estimation. Let us define the following edge-selection variables

10 $$\begin{array}{*{20}l} \beta(i,j) = \left\{\begin{array}{ll} 1 & \exists \ \text{edge between} \ i,j\\ 0 & \text{no edge} \end{array}\right.  \\ \quad \forall i,j \in \mathcal{G}:j>i   \end{array} $$

where *β*(*i*,*j*)=1 denotes that there is an edge between genes *i*,*j* in DAG $\mathcal {D}$ and *β*(*i*,*j*)=0 denotes that there exists no edge between genes *i* and *j*. Note that unlike *α*_*k*_(*i*,*j*)=1 for $k \in \mathcal {K}$, *β*(*i*,*j*)=1 does not capture directionality information about the graph topology, i.e., *β*(*i*,*j*)=1 states that there is an edge between genes *i*,*j* in DAG $\mathcal {D}$ without specifying the hierarchy among both genes. The topology $\mathcal {E}_{\mathcal {D}}$ of any DAG $\mathcal {D}$ can be represented by the corresponding set of class-selection variables $A^{\mathcal {D}} =\bigcup \limits _{i,j} \left \{ \alpha _{1}^{\mathcal {D}}(i,j),\ldots, \alpha _{5}^{\mathcal {D}}(i,j) \right \}$ together with the corresponding set of undirected edges {*β*(*i*,*j*)} for all $i,j:\in \mathcal {G}: j>i$. The set {*β*(*i*,*j*)} can be viewed as the undirected “skeleton” of the DAG that is represented by its corresponding set of class-selection variables $A^{\mathcal {D}}$. The GI-GENIE algorithm yields an estimate $\mathcal {E}_{\text {GI}}$ of the true DAG topology $\mathcal {E}_{\mathcal {D}}$ by computing sets $\mathrm {A}^{\mathrm {O}_{\text {GI-GENIE} }}$ and $ \left \{ \hat {\beta }(i,j) \right \}$ which are estimates of the true set of class-selection variables and edge-selection variables, $A^{\mathcal {D}}$ and {*β*(*i*,*j*)}, respectively. Based on SK, DK, and GI-profile data, the proposed GI-GENIE algorithm is formulated as the following LIP:

11a $$\begin{array}{*{20}l} \mathrm{O}_{\text{GI-GENIE}}: &  \\ \min_{\left\{ \alpha_{k}(i,j), \beta(i,j), z_{l}(i,j) \right\}} \; & \quad \lambda_{d} \sum\limits_{i=1}^{G} \sum\limits_{j=i+1}^{G} \left(\sum\limits_{k=1}^{\left| \mathcal{K} \right|} s_{k}(i,j)\alpha_{k}(i,j) \right)  \\ & - \lambda_{c} \sum\limits_{i=1}^{G} \sum\limits_{j=i+1}^{G} \rho(i,j)\beta(i,j)  \\ &+ \lambda_{p} \sum\limits_{i=1}^{G} \sum\limits_{j=i+1}^{G} \beta(i,j)  \end{array} $$

11b $$\begin{array}{*{20}l} \text{s. t.:} \; & \text{Eqs}.~(\text{4b})-~(\text{4d}) \end{array} $$

11c $$\begin{array}{*{20}l} & \beta(i,j) \in \left\{0,1 \right\}  \\ & \forall i,j \in \mathcal{G}: j>i \end{array} $$

11d $$\begin{array}{*{20}l} & z_{l}(i,j) \in \left\{0,1 \right\} \ \forall l \in \mathcal{G} \setminus \left\{i,j\right\},  \\ & \forall i,j \in \mathcal{G}: j>i \end{array} $$

11e $$\begin{array}{*{20}l} & 1- \alpha_{3}(i,j) \geq \beta(i,j)  \\ & \forall i,j \in \mathcal{G}: j>i  \end{array} $$

11f $$\begin{array}{*{20}l} & \mathcal{L}_{c} \Longrightarrow \ \text{additional topology} \\ & \text{constraints}  \end{array} $$

11g $$\begin{array}{*{20}l} & \left| \mathcal{G} \right|-2+ \beta(i,j) \geq \\ & 1+ \sum\limits_{l \in \mathcal{G} \setminus \left\{ i,j\right\}} z_{l}(i,j)  \end{array} $$

$$\begin{array}{*{20}l} & \forall i,j \in \mathcal{G}: j>i  \end{array} $$

where the scalars *λ*_*d*_,*λ*_*s*_,*λ*_*c*_, and *λ*_*p*_ are non-negative weighting constants to balance the impact of the SK and DK measurements and the GI-profile data, respectively, on the estimates. In particular, the parameter *λ*_*d*_ is used for dual purpose: (i) to scale the domain of the knockout scores *s*_*k*_(*i*,*j*) to the range [0,…,1] which is comparable to range of the correlation data *ρ*(*i*,*j*) and (ii) to trade-off the impact of the knockout scores *s*_*k*_(*i*,*j*) on the estimation outcome. The parameters *λ*_*c*_ and *λ*_*p*_ are in the interval [0,1] where *λ*_*c*_≥*λ*_*p*_. The GI-profile (GIP) term is given by

12 $$\begin{array}{*{20}l} - \lambda_{c} \sum\limits_{i=1}^{G} \sum\limits_{j=i+1}^{G} \rho(i,j)\beta(i,j) + \lambda_{p} \sum\limits_{i=1}^{G} \sum\limits_{j=i+1}^{G} \beta(i,j).  \end{array} $$

The quotient of $\frac {\lambda _{c}}{\lambda _{p}}$ defines the threshold for reward of the GI-profile (GIP) term in Eq. (12), where setting the edge selection variable *β*(*i*,*j*)=1 is rewarded if the corresponding GI-profile measurement *ρ*(*i*,*j*) is above the quotient $\frac {\lambda _{c}}{\lambda _{p}}$.

The auxiliary variables $z_{l}(i,j) \forall i,j,l \in \mathcal {G}: j>i, l\neq i, l\neq j$ are generally necessary to ensure that the information about the topology of DAG $\mathcal {D}$, which is encoded in the pattern of selection variables $\mathrm {A}^{\mathrm {O}_{\text {GI-GENIE} }}$ detected by program O_GI-GENIE_, is not contradicting with the set of edge selection variables $ \left \{ \hat {\beta }(i,j) \right \} \, \forall i,j \in \mathcal {G}:j>i$ detected by program O_GI-GENIE_. In particular, given that the detected pattern of selection variables $\mathrm {A}^{\mathrm {O}_{\text {GI-GENIE} }}$ enforces that there is an edge between genes *i*,*j* in DAG $\mathcal {D}$, then the auxiliary variables ensure that the corresponding edge selection variable indicates that there is an edge between genes *i*,*j*, i.e., $\hat {\beta }(i,j) = 1$. Furthermore, given that the detected pattern of selection variables $\mathrm {A}^{\mathrm {O}_{\text {GI-GENIE} }}$ enforces that there is no edge between genes *i*,*j* in DAG $\mathcal {D}$, then the auxiliary variables ensure that the corresponding edge selection variable indicates that there is no edge between genes *i*,*j*, i.e., $\hat {\beta }(i,j) = 0$. On the contrary, assume that the detected edge selection variables enforce that there is an edge between genes *i*,*j* in DAG $\mathcal {D}$, i.e., $\hat {\beta }(i,j) = 1$, then the *z*_*l*_(*i*,*j*) ensure that the detected pattern of selection variables $\mathrm {A}^{\mathrm {O}_{\text {GI-GENIE} }}$ must fulfill one of the conditions stated in Table 1. Consequently, given that the detected edge selection variables enforce that there is no edge between genes *i*,*j* in DAG $\mathcal {D}$, i.e., $\hat {\beta }(i,j) = 0$, then the *z*_*l*_(*i*,*j*) ensure that the detected pattern of selection variables $\mathrm {A}^{\mathrm {O}_{\text {GI-GENIE} }}$ does not fulfill any of the conditions stated in Table 1.

Furthermore, let the auxiliary parameters

13 $$\begin{array}{*{20}l} q(i,j) = \left\{\begin{array}{ll} 1 & \rho(i,j) \geq \frac{\lambda_{c}}{\lambda_{p}} \\ 0 & \rho(i,j) < \frac{\lambda_{c}}{\lambda_{p}} \end{array}\right.  \\ \forall \, i,j \in \mathcal{G}:j>i \end{array} $$

describe the detection of the edges of DAG $\mathcal {D}$ based on GI-profile data only, where *q*(*i*,*j*)=1 denotes that there is an edge between genes *i*,*j* and *q*(*i*,*j*)=0 denotes that there is no edge between genes *i*,*j*. Since any pattern of hierarchical relationship classes implies a specific set of edges and any set of edges implies a specific pattern of hierarchical relationship classes, there is a strong coupling of constraints, i.e., there are strong logical implications among the selection variables *α*_*k*_(*i*,*j*) and the selection variables *β*(*i*,*j*); the constraints in Eqs. (11e) to (11g) ensure that the detected hierarchical relationship classes and the corresponding edges, i.e., the *α*_*k*_(*i*,*j*) and *β*(*i*,*j*), are not mutually contradicting. Given that two genes *i*,*j* in DAG $\mathcal {D}$ are independent, i.e., *α*_3_(*i*,*j*)=1, there cannot exist an edge between those genes in DAG $\mathcal {D}$, i.e., *β*(*i*,*j*)=0. This logical implication is reflected by (11e). Set $\mathcal {L}_{c}$ in (11f) and the linear integer inequalities in (11g) model conditions E_1_ to E_4_ of our proposed edge detection policy as stated in Table 1. Since we do not want to redundantly expand the set of selection variables *α*_*k*_(*i*,*j*) to all $i,j \in \mathcal {G}: j \neq i$ in order to not increase the complexity of program O_GI-GENIE_, we have to consider three cases when formulating conditions E_1_ to E_4_ of Table 1 as linear integer inequalities, i.e., $i,j,l \in \mathcal {G}: l>j>i$, $i,j,l \in \mathcal {G}: j>i>l$ and $i,j,l \in \mathcal {G}: j>l>i$. Then, the constraints in set $\mathcal {L}_{c,1}$

14a $$\begin{array}{*{20}l} \mathcal{L}_{c,1} = \left\{ {\vphantom{\frac{0}{0}}}\right.& \\ & 1 -\beta(i,j) \geq \alpha_{1}(i,j) + \alpha_{1}(i,l) + \alpha_{2}(j,l) -2  \end{array} $$

14b $$\begin{array}{*{20}l} & \frac{1}{2} \left(\alpha_{1}(i,l) + \alpha_{2}(j,l) \right) \geq \alpha_{1}(i,j) - z_{l}(i,j)  \end{array} $$

14c $$\begin{array}{*{20}l} & 1 -\beta(i,j) \geq \alpha_{2}(i,j) + \alpha_{2}(i,l) + \alpha_{1}(j,l) -2 \\ & \frac{1}{2} \left(\alpha_{2}(i,l) + \alpha_{1}(j,l) \right) \geq \alpha_{2}(i,j) - z_{l}(i,j)  \end{array} $$

14d $$\begin{array}{*{20}l} & 1 -\beta(i,j) + q(i,j) \geq \alpha_{4}(i,j) + \alpha_{1}(i,l) +  \\ & \alpha_{4}(i,l) + \alpha_{2}(j,l) +\alpha_{5}(j,l) -2  \end{array} $$

14e $$\begin{array}{*{20}l} & \frac{1}{2} \left(\alpha_{1}(i,l) + \alpha_{4}(i,l) + \alpha_{2}(j,l)+\alpha_{5}(j,l)\right) \geq  \\ & \alpha_{4}(i,j) - z_{l}(i,j) - q(i,j)  \end{array} $$

14f $$\begin{array}{*{20}l} & 2 -\beta(i,j) - q(i,j) \geq \alpha_{4}(i,j) + \alpha_{1}(i,l)  \\ & + \alpha_{2}(j,l) + \alpha_{5}(j,l) -2  \end{array} $$

14g $$\begin{array}{*{20}l} & \frac{1}{2} \left(\alpha_{1}(i,l) + \alpha_{2}(j,l)\right) \geq  \\ & \alpha_{4}(i,j) - z_{l}(i,j) -1+ q(i,j)  \end{array} $$

14h $$\begin{array}{*{20}l} & 1 -\beta(i,j) + q(i,j) \geq \alpha_{5}(i,j) + \alpha_{2}(i,l) +  \\ & \alpha_{5}(i,l) + \alpha_{1}(j,l) +\alpha_{4}(j,l) -2  \\ & \frac{1}{2} \left(\alpha_{2}(i,l) + \alpha_{5}(i,l) + \alpha_{1}(j,l)+\alpha_{4}(j,l) \right) \geq \\ & \alpha_{5}(i,j) - z_{l}(i,j) - q(i,j)  \end{array} $$

14i $$\begin{array}{*{20}l} & 2 -\beta(i,j) - q(i,j) \geq \alpha_{5}(i,j) + \alpha_{2}(i,l)  \\ & + \alpha_{5}(i,l) + \alpha_{1}(j,l) -2  \\ & \frac{1}{2} \left(\alpha_{2}(i,l) + \alpha_{1}(j,l) \right) \geq  \\ & \alpha_{5}(i,j) - z_{l}(i,j) -1+ q(i,j)  \end{array} $$

$$\begin{array}{*{20}l} & \left.{\vphantom{\frac{0}{0}}}\right\} \forall i,j,l \in \mathcal{G}: l>j>i  \end{array} $$

model the logical implications among the selection variables $\phantom {\dot {i}\!}\alpha _{k}(i,j), \alpha _{k'}(i,l), \alpha _{k^{\prime \prime }}(j,l)$, and *β*(*i*,*j*) for $\ k,k^{\prime }, k^{\prime \prime } \in \mathcal {K}, \forall i,j,l \in \mathcal {G}: l>j>i$. Together with (11g), constraints (14a)–(14b) model condition E_1_ of our detection policy taking into account the GI-profile information *ρ*(*i*,*j*) via selection variables *β*(*i*,*j*). Assume that based on the SK and DK phenotypes, it is most consistent that *α*_1_(*i*,*j*)=*α*_1_(*i*,*l*)=*α*_2_(*j*,*l*)=1 for at least one gene *l* in DAG $\mathcal {D}$ which corresponds to condition E_1_ being violated. Hence, there cannot exist an edge between genes *i* and *j* in DAG $\mathcal {D}$. In this case, the RHS of (14a) amounts to 1 which enforces the LHS of (14a) to amount to 1 as well, i.e., *β*(*i*,*j*)=0. Note that for *α*_1_(*i*,*j*)=*α*_1_(*i*,*l*)=*α*_2_(*j*,*l*)=1, (14b) makes no restrictions on *z*_*l*_(*i*,*j*). Furthermore, assume that for genes *i*,*j*, based on the SK and DK phenotypes, it is most consistent that *α*_1_(*i*,*j*)=1, but *α*_1_(*i*,*l*) and *α*_2_(*j*,*l*) are not jointly 1 for all other genes $l \in \mathcal {G}: l>j>i $, i.e., *α*_1_(*i*,*l*)+*α*_1_(*j*,*l*)<2, then there is an edge between genes *i*,*j* in DAG $\mathcal {D}$ according to condition E_1_. In this case, it is obvious that (14a) is always fulfilled, i.e., there are no restrictions on *β*(*i*,*j*) by (14a). Since *α*_1_(*i*,*j*)=1 and *α*_1_(*i*,*l*)+*α*_2_(*j*,*l*)≤1 for all $l \in \mathcal {G}: l>j>i $, constraint (14b) can only be fulfilled if $z_{l}(i,j) =1 \ \forall l \in \mathcal {G}: l>j>i$. Hence, this enforces *β*(*i*,*j*)=1 due to constraint (11g). In this case, constraint (14b) forces $z_{l}(i,j)=1 \ \forall l \in \mathcal {G}:l > j >i$. Hence, given that $z_{l}(i,j)=1 \ \forall l \in \mathcal {G}:l > j >i$, constraint (11g) sets *β*(*i*,*j*)=1.

Given that the GI-profile data strongly supports that there is no edge between genes *i*,*j* in DAG $\mathcal {D}$, i.e., *β*(*i*,*j*)=0, and *α*_1_(*i*,*j*)=1 is most consistent based on the SK and DK phenotypes measured, then it follows from (11g) that there must be at least one $l \in \mathcal {G}: l > j>i$ for which *z*_*l*_(*i*,*j*)=0. In this case, with *β*(*i*,*j*)=0, *α*_1_(*i*,*j*)=1, and *z*_*l*_(*i*,*j*)=0, the RHS of (14b) amounts to 1, forcing the LHS of (14b) to amount to 1 as well, i.e., *α*_1_(*i*,*l*)=1 and *α*_2_(*j*,*l*)=1, which is together with the assumption of *α*_1_(*i*,*j*)=1 a combination that violates the existence of a direct edge between genes *i* and *j*. Furthermore, note that (14a) does not have any implications on the selection variables *α*_1_(*i*,*j*),*α*_1_(*i*,*l*), and *α*_2_(*j*,*l*) for the case that *β*(*i*,*j*)=0 and *z*_*l*_(*i*,*j*)=0.

Assume that the GI-profile data strongly supports that there is an edge between genes *i*,*j* in DAG $\mathcal {D}$, i.e., *β*(*i*,*j*)=1, and *α*_1_(*i*,*j*)=1 is most consistent based on the SK and DK phenotypes measured, then according to (14a), there cannot be any gene $l \in \mathcal {G}: l >j>i$ for which *α*_1_(*i*,*l*)=1 and *α*_2_(*j*,*l*)=1. Hence, Eq. (14b) can only be fulfilled if $z_{l}(i,j)=1 \ \forall l \in \mathcal {G}:l>j>i$. Thus, (11g) is fulfilled with equality. We remark that given *α*_1_(*i*,*j*)=1, constraints (14c) to (14i) are always fulfilled, i.e., they do not pose any implications among the selection variables *α*_*k*_(*i*,*j*) and *β*(*i*,*j*). Together with (11g), the two inequalities in (14c) model condition E_3_ where we can elucidate their functionality in the same fashion as before. Constraints (14d) to (14g) along with (11g) model a minor modification of condition E_2_ where we detect not only all necessary edges but also optional edges given that their existence is strongly supported by the GI-profile. Given that the existence of an edge between genes *i*,*j* in DAG $\mathcal {D}$ is not strongly supported by the GI-profile, i.e., *q*(*i*,*j*)=0, constraints (14d) to (14e) along with (11g) model condition E_2_ which only allows necessary edges to be detected and we can elucidate their functionality in the same fashion as in (14a) to (14b). Note that (14f) to (14g) are always fulfilled for *q*(*i*,*j*)=0, i.e., no implications among the selection variables *α*_*k*_(*i*,*j*) and *β*(*i*,*j*) are posed. Assuming that the existence of an edge between genes *i*,*j* in DAG $\mathcal {D}$ is strongly supported by the GI-profile, i.e., *q*(*i*,*j*)=1, then the constraints in (14d) and (14e) are always fulfilled, i.e., no implications among the selection variables *α*_*k*_(*i*,*j*) and *β*(*i*,*j*) are posed by (14d) and (14e). However, constraints (14f) and (14g) pose relaxed logical implications among the selection variables *α*_*k*_(*i*,*j*) and *β*(*i*,*j*) compared to constraints (14d) to (14e). Hence, given that *q*(*i*,*j*)=1 and *α*_4_(*i*,*j*)=1, an edge between genes *i*,*j* in DAG $\mathcal {D}$ is detected if it is allowed by the pattern of hierarchical relationship classes. Constraints (14h) to (14i) along with (11g) model a minor modification of condition E_4_ where we detect not only all necessary edges but also optional edges given that their existence is strongly supported by the GI-profile. Furthermore, the functionality of constraints (14h) to (14i) can be explained with the same line of argument as used to elucidate constraints (14d) to (14g).

Denote $\mathcal {L}_{c,2}$ and $\mathcal {L}_{c,3}$ as the sets of topology constraints that model the logical coupling among the selection variables $\phantom {\dot {i}\!}\alpha _{k}(i,j), \alpha _{k^{\prime }}(i,l),\alpha _{k^{\prime \prime }}(j,l)$, and *β*(*i*,*j*) for $k, k^{\prime }, k^{\prime \prime } \in \mathcal {K}$ and $i,j,l \in \mathcal {G}: j>i>l$ and $i,j,l \in \mathcal {G}: j>l>i$, respectively. Then, the full set of coupled constraints of the selection variables *α*_*k*_(*i*,*j*) and *β*(*i*,*j*) is given by

15 $$\begin{array}{*{20}l} \mathcal{L}_{c} = \bigcup\limits_{\kappa=1}^{3} \left\{ \mathcal{L}_{c,\kappa} \right\}  \end{array} $$

where we again refer the interested reader to [30] for a detailed description of $\mathcal {L}_{c}$. We obtain an estimate $\mathcal {E}_{\text {GI}}$ of the true set of edges $\mathcal {E}_{\mathcal {D}}$ of DAG $\mathcal {D}$ based on the computed set of edge selection variables $ \left \{ \hat {\beta }(i,j) \right \}$ of program O_GI-GENIE_ where we infer the directionality of the edges according to $\mathrm {A}^{\mathrm {O}_{\text {GI-GENIE}}}$. Note that all reporter node edges are computed according to our proposed reporter node edge detection policy as given in Table 2. Since the reporter node is an artificial node in the concept of a DAG, there is no GI-profile data $\rho (i,R) \, \forall i \in \mathcal {G}$ and thus, no edge selection variable $\beta (i,R) \, \forall i \in \mathcal {G}$ according to (10).

## Sequential scalability technique

Due to the combinatorial nature of problems O_GENIE_ and O_GI-GENIE_, the GENIE algorithm and GI-GENIE algorithm, respectively, cannot be applied to the data of large sets of genes $\mathcal {G}$, since the number of candidate solutions to problems O_GENIE_ and O_GI-GENIE_, respectively, grows exponentially with the number of genes. In order to nevertheless obtain statistically significant statements about the interactions among genes in a large set of genes $\mathcal {G}$, we propose the sequential scalability (SEQSCA) technique which is based on the GENIE algorithm and the GI-GENIE algorithm, respectively.

In particular, we create a sequence of *S* subsets $\left \{ \mathcal {G}_{s} \right \}_{1}^{S}$ of the full set of genes $\mathcal {G}$, i.e., $\mathcal {G}_{s} \subset \mathcal {G},~\text {and}~ \ \forall s \in \left \{1,\ldots,S \right \}$, where we estimate the topology $\mathcal {E}_{\mathcal {D},s}$ of each DAG $\mathcal {D}_{s}$, underlying the data of the subset of genes $\mathcal {G}_{s}$, by the GENIE or GI-GENIE algorithm, respectively, in order to translate the estimated topology $\mathcal {E}_{\mathcal {D},s}$ of DAG $\mathcal {D}_{s}$ into the corresponding adjacency matrix ***M***_*s*_ for each *s*∈{1,…,*S*}. Based on the computed sequence of adjacency matrices $\left \{ \boldsymbol {M}_{s} \right \}_{1}^{S}$, we iteratively compute the reliability matrix ***M***∈[0,1]^*N*×*N*^ of the full set of genes $\mathcal {G}$ in such a way that each entry $\left [ \boldsymbol {M} \right ]_{i,j \in \mathcal {G}}$ describes the empirical probability of an edge to exist between genes $i,j \in \mathcal {G}$, i.e., the empirical probability that genes $i,j \in \mathcal {G}$ directly interact with each other, where a value of 0 means that there is an interaction between the considered pair of genes with probability 0 and a value of 1 means that the considered pair of genes interacts with probability 1.

In particular, in each iteration *s*, we consider a subset $\mathcal {G}_{s}$ of size $N_{S} \ll \left | \mathcal {G} \right |$ of the full set of genes $\mathcal {G}$, where each gene of $\mathcal {G}_{s}$ is selected from $\mathcal {G}$ without replacement with equal probability. Based on the selected subset $\mathcal {G}_{s}$, we compute in each iteration *s* an estimate $\mathcal {E}_{\mathcal {D},s}$ of the true topology of DAG $\mathcal {D}_{s}$, underlying the observed data of the genes in subset $\mathcal {G}_{s}$, by the GENIE or the GI-GENIE algorithm, respectively. Furthermore, the topology estimate $\mathcal {E}_{\mathcal {D},s}$ of DAG $\mathcal {D}_{s}$ is translated into the corresponding adjacency matrix ***M***_*s*_. The update of the reliability matrix for iteration *s* is computed according to Eq. (16)

16 $$\begin{array}{*{20}l} \left[ \boldsymbol{M}^{(s+1)} \right]_{i,j} = \left[ \boldsymbol{M}^{(s)} \right]_{i,j} + \left[ \boldsymbol{M}_{s} \right]_{\kappa_{i}, \kappa_{j}} \quad \forall i,j \in \mathcal{G}_{s}  \end{array} $$

with ***M*** ^(*s*)^ being the *N*×*N* reliability matrix at iteration *s*, $\kappa _{i} \in \left \{1,\ldots, N_{S} \right \} \ \forall i \in \mathcal {G}_{s}$, ∪_*i*_ *κ*_*i*_={1,…,*N*_*S*_} and *κ*_*i*_<*κ*_*j*_ for all *i*<*j*. Finally, we obtain the reliability matrix ***M*** of the full set of genes $\mathcal {G}$ by normalizing each entry $ \left [ \boldsymbol {M}^{(S)} \right ]_{i,j} \ i,j \in \mathcal {G}$ by *n*_*i*,*j*_ that is the frequency of how often detecting an edge between genes *i* and *j* has been considered. Note that the proposed SEQSCA technique does not intend to yield valid DAGs but to provide statistical statements to which empirical probability two genes interact with each other.

In Table 3, we have summarized the SEQSCA technique. Finally, by means of the SEQSCA technique, we are able to provide statistically significant statements about the interactions among the genes from a large set $\mathcal {G}$ by using the GENIE or GI-GENIE algorithm, respectively, in a sequential fashion.

**Table 3:** Summary of the proposed SEQSCA-algorithm

**Initialization:** ***M*** ^(0)^=***0***_*N*×*N*_; $\phantom {\dot {i}\!}\boldsymbol {M}_{s=0} = \boldsymbol {0}_{N_{S} \times N_{S}}$; frequency counter $n^{(0)}_{i,j} = 0$	
**Repeat:**	
1: Select subset $\mathcal {G}_{s}$ of size *N*_*S*_ from $\mathcal {G}$; draw each gene from $\mathcal {G}$ with equal probability without replacement	
2: Update: $n^{(s+1)}_{i,j} = n^{(s)}_{i,j} + 1$ for all $i,j \in \mathcal {G}_{s}$	
3: Estimate the DAG topology $\mathcal {E}_{s}$ of set $\mathcal {G}_{s}$ using GENIE, GI-GENIE, respectively; ⇒***M***_*s*_	
4: Update reliability matrix ***M*** ^(*s*)^ according to Eq. (16)	
7: Update iteration number: *s*←*s*+1	
**Until:** *s*=*S*;	
Set $ \left [ \boldsymbol {M} \right ]_{i,j} = \left [ \boldsymbol {M}^{(S)} \right ]_{i,j} / n^{(S)}_{i,j} \, \forall i,j \in \mathcal {G}$	

## Simulation results

In this section, we first demonstrate the performance of the GENIE algorithm and the GI-GENIE algorithm with respect to conventional techniques for simulated data and further provide statistically significant statements on the interactions among the genes from a large set of genes based on real data using the proposed SEQSCA technique. For the implementation of the proposed algorithms, we used the popular CVX interface [31] along with the well-known MOSEK solver [32].

### Synthetic data results

We have generated the ideal SK phenotypes $R(i) \in \mathbb {R}$ for all $i \in \mathcal {G}$ as well as the ideal DK phenotypes $R(i,j) \in \mathbb {R}$ for all $i,j \in \mathcal {G}: j>i$ according to the model of [2] as displayed in Fig. 2, where we have corrupted the ideal SK and DK phenotypes by independently and identically distributed zero-mean Gaussian noise with variance *σ* ^2^. Furthermore, the GI-profile data $\rho (i,j) \forall i,j \in \mathcal {G}: j>i$ has been generated on the basis of the SK and DK phenotypes. We compare both the GENIE algorithm and the GI-GENIE algorithm with the well-known GI-profile approach [2, 33], where the Pearson correlation between the GI-profiles of genes *i* and *j* is computed and an edge in the DAG is detected if the Pearson correlation is above a pre-defined threshold *t*_corr_, where the directionality is inferred from the selection variable *α*_*k*_(*i*,*j*) corresponding to the least mismatch model *μ*_*k*_(*i*,*j*). Furthermore, we compare our proposed methods with the solution of program O_GENIE_ without considering set $\mathcal {L}$ as a constraint, which means simply classifying each pair *i*,*j* to the least mismatch scoring hierarchical relationship class based on the SK and DK phenotypes *R*(*i*) and *R*(*i*,*j*), respectively, without ensuring that the resulting pattern of hierarchical relationship classes represents a valid DAG.

In order to limit the Monte Carlo simulation time, we consider a total of 10 genes amounting to 225 binary variables and 2670 constraints for the GENIE algorithm and 630 binary variables and 9645 constraints for the GI-GENIE algorithm, respectively. For the GENIE method without considering the consistency constraints in $\mathcal {L}$, we have 225 binary variables and 270 constraints. Since we infer the edge orientation for the Pearson correlation-based method from the least mismatch scoring model, i.e., from the GENIE method without considering the consistency constraints in $\mathcal {L}$, we have 270 binary variables and 270 constraints.

**Fig. 7:**
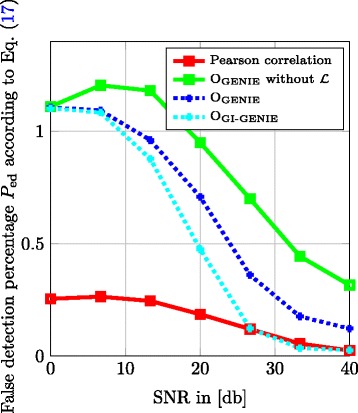
*D*_ed_ versus SNR; *t*_corr_=0.6; 200 Monte Carlo runs; *λ*_*d*_=0.05, *λ*_*c*_=1, *λ*_*p*_=0.8

In Fig. 7, we display the false detection percentage of edges *P*_ed_ in the detected DAG normalized to the true number of edges $\left | \mathcal {E}_{\mathcal {D}} \right |$ as defined in Eq. (17) versus the SNR.

17 $$\begin{array}{*{20}l} P_{\text{ed}} = \frac{\left| \left(\mathcal{E}_{\mathcal{D}} \bigcup \hat{\mathcal{E}}_{\mathcal{D}} \right) \setminus \mathcal{E}_{\mathcal{D}} \right| }{\left| \mathcal{E}_{\mathcal{D}} \right|}  \end{array} $$

**Fig. 8:**
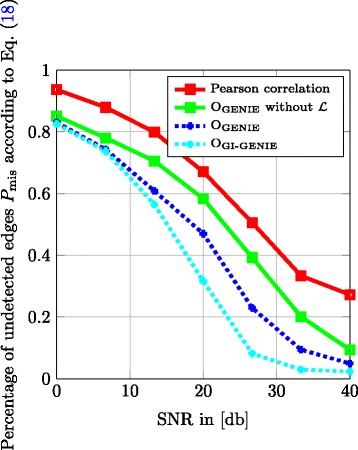
*D*_mis_ versus SNR; *t*_corr_=0.6; 200 Monte Carlo runs; *λ*_*d*_=0.05, *λ*_*c*_=1, *λ*_*p*_=0.8

In Fig. 8, we display the percentage of undetected edges *P*_mis_ in the detected DAG normalized to the true number of edges $\left | \mathcal {E}_{\mathcal {D}} \right |$ as defined in Eq. (18) versus the SNR, i.e.,

18 $$\begin{array}{*{20}l} P_{\text{mis}} = \frac{\left| \left(\mathcal{E}_{\mathcal{D}} \bigcup \hat{\mathcal{E}}_{\mathcal{D}} \right) \setminus \hat{\mathcal{E}}_{\mathcal{D}} \right|}{\left| \mathcal{E}_{\mathcal{D}} \right|}  \end{array} $$

Note that in multi-hypothesis testing problems, it is common to view the diagnostic plots in Figs. 7 and 8 jointly to assess the quality of the proposed algorithms. In Fig. 7, we observe that in the low SNR regime, the Pearson correlation-based method performs best in terms of false detection percentage of edges *P*_ed_; however, it fails to improve performance with increasing SNR, because for correct directionality information of the edges, this approach relies on the hierarchical relationship classes detected by method O_GENIE_ without considering $\mathcal {L}$. Especially in the high SNR regime, the proposed GENIE and GI-GENIE methods clearly outperform program O_GENIE_ without the topology rule set $\mathcal {L}$ and approach and respectively reach the performance of the Pearson correlation method. However, the very good performance of the Pearson correlation method in terms of false detection percentage of edges *P*_ed_ according to Eq. (17) comes at the cost of a rather poor performance in terms of the percentage of undetected edges *P*_mis_ according to Eq. (18) as can be seen in Fig. 8. In particular, in terms of the percentage of undetected edges *P*_mis_, all of the proposed methods outperform the Pearson correlation method. Note that in the high SNR regime, the GI-GENIE of combining SK, DK, and GI-profile data yields the best of both worlds, i.e., it shows an equivalent performance as the Pearson correlation method in terms of false detection percentage of edges *P*_ed_, as well as an improvement of the strong performance of the GENIE method in terms of the percentage of undetected edges *P*_mis_.

### Real data results

**Fig. 9:**
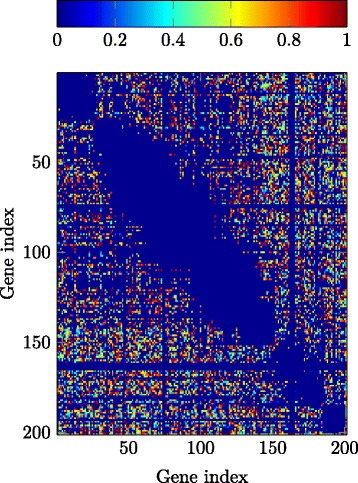
Reliability matrix ***M***_G_; *S*=5*e* ^4^ subsets considered; subset size *N*_*S*_=10

**Fig. 10:**
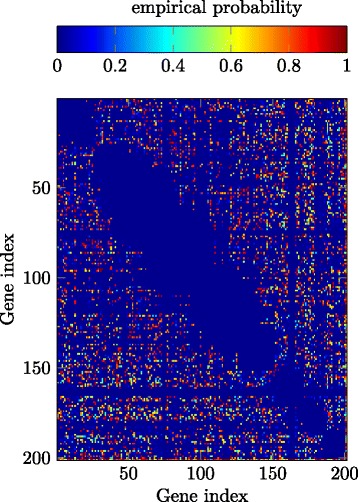
Reliability matrix ***M***_GI_; *S*=5*e* ^4^ subsets considered; subset size *N*_*S*_=10; *λ*_*d*_=1*e*3, *λ*_*c*_=1, *λ*_*p*_=0.85

Since discovering genetic interaction maps, i.e., DAGs, for specific organisms is an ongoing field of research and the knowledge on genetic interactions is far away from being complete, there is generally no *ground truth* to directly compare with, even not for yeast which is one of the best understood organisms in this aspect. Therefore, we base the evaluation of the detection performance of the GENIE and the GI-GENIE methods on the biological knowledge that genetic interactions are generally rare and furthermore on the successful detection of known interactions provided by the well-known *yeast database* of [34]. We remark that to obtain statistically significant statements about large sets of genes, we have applied the proposed GENIE and GI-GENIE algorithms, respectively, along with the SEQSCA technique presented above. To demonstrate the benefit of using multiple data types instead of only one data type, we compare the reliability matrix results for SEQSCA and GI-GENIE with SEQSCA and GENIE which only utilizes SK/DK data. We have applied the abovementioned algorithms to the dataset reported in [35] to obtain the reliability matrices for the GENIE-based SEQSCA as well as for the GI-GENIE-based SEQSCA, ***M***_G_ and ***M***_GI_, respectively. The phenotypes reported in [35] are colony size measurements normalized to the wild-type size for each particular SK and DK, respectively. Typically, the colony size serves as a proxy for the fitness of the organism under study, which is the actual cell function of interest that cannot be observed. Therefore, the phenotypes in [35] are non-negative real numbers within the range [0,*C*_max_], where *C*_max_ denotes the maximum size dictated by the experiment setup. For computational reasons, we only considered the first 200 genes, i.e., $\left | \mathcal {G} \right | = 200$, of the *query gene list* of [35]. Figure 9 shows ***M***_G_ obtained by the GENIE-based SEQSCA. In Fig. 10, we have displayed ***M***_GI_ obtained by the GI-GENIE-based SEQSCA. For both results, we decomposed $\mathcal {G}$ into a sequence of *S*=5*e*4 subsets $\mathcal {G}_{s}$ of equal size *N*_*s*_=10. In Fig. 9, 78*%* of the gene pairs *i*,*j* considered by ***M***_G_ of the GENIE-based SEQSCA interact with each other with an empirical probability of less than 20%, i.e., [***M***_G_]_*i*,*j*_≤.2. Hence, the GENIE-based SEQSCA yields approximately sparse results. This is a good performance in terms of sparsity, since it is known from biology that genetic interactions are generally very rare. However, we can clearly see by the reliability matrix ***M***_GI_ that the proposed GI-GENIE algorithm predicts genetic interactions with a much lower frequency as compared to the GENIE algorithm, which means a very good performance in terms of sparsity. We have computed the acceptance ratio

19 $$ \Gamma = \frac{N_{\mathrm{r}}}{N_{\mathrm{t}}}  $$

where *N*_r_ is the number of interactions found with high significance ([***M***_G_]_*i*,*j*_,[***M***_GI_]_*i*,*j*_≥1−*ε*) and which are deposited in the database of [34] as well. *N*_t_ is the total number of highly significant interactions. Given the confirmed interactions at [34] for our set of genes under study, we remark that evaluating the number of confirmed interactions, that we have also found with our proposed method, would not be a reasonable performance metric, since [34] combines knowledge and experimental results of numerous sources. In contrast to that, we only had the dataset of [35] which only considers a particular phenotype, i.e., colony growth. As depicted in Table 4, we have computed *Γ* for both the GI-GENIE-based SEQSCA and the GENIE-based SEQSCA. It is obvious that the GI-GENIE-based SEQCA outperforms the GENIE-based SEQSCA, since the acceptance ratio for the GI-GENIE-based SEQSCA is significantly higher than the one of the GENIE-based SEQSCA.

**Table 4:** Acceptance ratios; *ε*=0.05

Method:	*Γ* (*%*)
SEQSCA and GENIE	53
SEQSCA and GI-GENIE	74

## Conclusions

In this paper, we have considered the problem of learning the interactions between genes in a genetic network. We have proposed the GENIE algorithm and the GI-GENIE algorithm to reconstruct the DAG underlying the observed data. The GENIE method is purely based on SK and DK data whereas the GI-GENIE method combines SK and DK data with GI-profile data in order to compute an estimate of the true DAG topology. In Section 5, we have presented the SEQSCA technique in order to obtain statistically significant statements about the interactions among a large set of genes under study. Furthermore, we have shown by simulations that the GI-GENIE algorithm outperforms the conventional techniques and the GENIE algorithm due to the combination of multiple data types, i.e., SK/DK and GI-profile data. Finally, based on the SEQSCA technique, we have presented real data results for the GENIE and the GI-GENIE algorithm, respectively, where we have confirmed that the GI-GENIE method outperforms the GENIE method.

## Endnote

^1^ In a discrete optimization context, the class-selection variables defined in (3) are denoted as SOS-1 type variables. However, for the sake of readability, we will mostly omit this optimization context-based annotation and refer to the variables defined in (3) as class-selection variables.
